# Oncogenic viruses rewire the epigenome in human cancer

**DOI:** 10.3389/fcimb.2025.1617198

**Published:** 2025-06-10

**Authors:** Jhommara Bautista, Andrés Lopez-Cortes

**Affiliations:** ^1^ Cancer Research Group (CRG), Faculty of Medicine, Universidad de Las Américas, Quito, Ecuador; ^2^ Facultade de Ciencias, Campus de A Zapateira, Universidade da Coruña, A Coruña, Spain; ^3^ Instituto de Investigación Biomédica de A Coruña (INIBIC), Universidade da Coruña, A Coruña, Spain

**Keywords:** oncogenic virus, oncolytic virotherapy, epigenome, cancer, immune modulation, drugs

## Abstract

Viruses contribute to approximately 15–20% of global cancer cases, yet the full spectrum of their oncogenic mechanisms continues to be uncovered. Beyond the classical roles of genome integration, chronic inflammation, and immune evasion, mounting evidence reveals that oncogenic viruses—including the human papillomavirus (HPV), Epstein–Barr virus (EBV), hepatitis B virus (HBV), hepatitis C virus (HCV), and Human T-cell leukemia virus type 1 (HTLV-1)—profoundly reshape the host epigenome to establish persistent infection and promote tumorigenesis. These viruses orchestrate widespread and durable changes in DNA methylation, histone modification, chromatin accessibility, and non-coding RNA expression, silencing tumor suppressors, deregulating oncogenic pathways, and inducing stemness-like phenotypes. In this review, we provide a comprehensive synthesis of how distinct oncogenic viruses modulate the epigenetic landscape across tissue contexts, with a focus on cervical, hepatic, and lymphoepithelial cancers. We also explore how these virus-induced epigenetic “scars” may persist after viral clearance and highlight recent advances in therapeutic targeting. Emerging therapeutic strategies that integrate oncolytic virotherapy, epigenetic drugs, and immune modulation through combinational therapy offer synergistic mechanisms to overcome immune resistance and epigenetic silencing in virus-induced cancers. These integrated approaches hold transformative potential for more durable and targeted treatment outcomes.

## Introduction

A substantial proportion of human cancers—estimated at approximately 15–20% globally—are attributable to viral infections, with oncogenic viruses playing a pivotal role in tumor initiation and progression through multifaceted mechanisms (Ameya and Birri, 2023; Poreba et al., 2011; Tashiro and Brenner, 2017). These viruses include both DNA and RNA types, such as human papillomavirus (HPV), Epstein–Barr virus (EBV), hepatitis B and C viruses (HBV, HCV), and human T-cell lymphotropic virus (HTLV-1), which have been linked to a range of malignancies including cervical, liver, nasopharyngeal, and hematological cancers​ (Poreba et al., 2011; Ameya and Birri, 2023; Damian, 2025).

Oncogenic viruses contribute to carcinogenesis through a combination of direct and indirect mechanisms. These include integration into the host genome, disruption of tumor suppressor pathways, sustained expression of viral oncoproteins, chronic inflammation, and immune evasion (Poreba et al., 2011; Tashiro and Brenner, 2017; Szewczyk-Roszczenko et al., 2025). Importantly, recent advances have revealed that these viruses also exploit host epigenetic machinery to promote cellular transformation. By modulating DNA methylation, histone modifications, chromatin remodeling, and non-coding RNA expression, oncogenic viruses reprogram the host epigenome to favor viral persistence and oncogenesis (Sultan et al., 2025; Poreba et al., 2011; Flanagan, 2007).

These epigenetic alterations are not merely bystanders of transformation; they actively disrupt normal gene regulation and cellular identity. For instance, hypermethylation of tumor suppressor gene promoters and histone deacetylation facilitate immune escape and uncontrolled cell proliferation (Flanagan, 2007; Sultan et al., 2025). Moreover, persistent viral infections are often accompanied by global hypomethylation, contributing to genomic instability—a hallmark of cancer​ (Poreba et al., 2011; Szewczyk-Roszczenko et al., 2025). Intriguingly, some viruses, such as HPV and EBV, encode proteins that directly interact with epigenetic regulators, including histone acetyltransferases and DNA methyltransferases, to drive these changes (Poreba et al., 2011; Flanagan, 2007).

**Figure 1:**
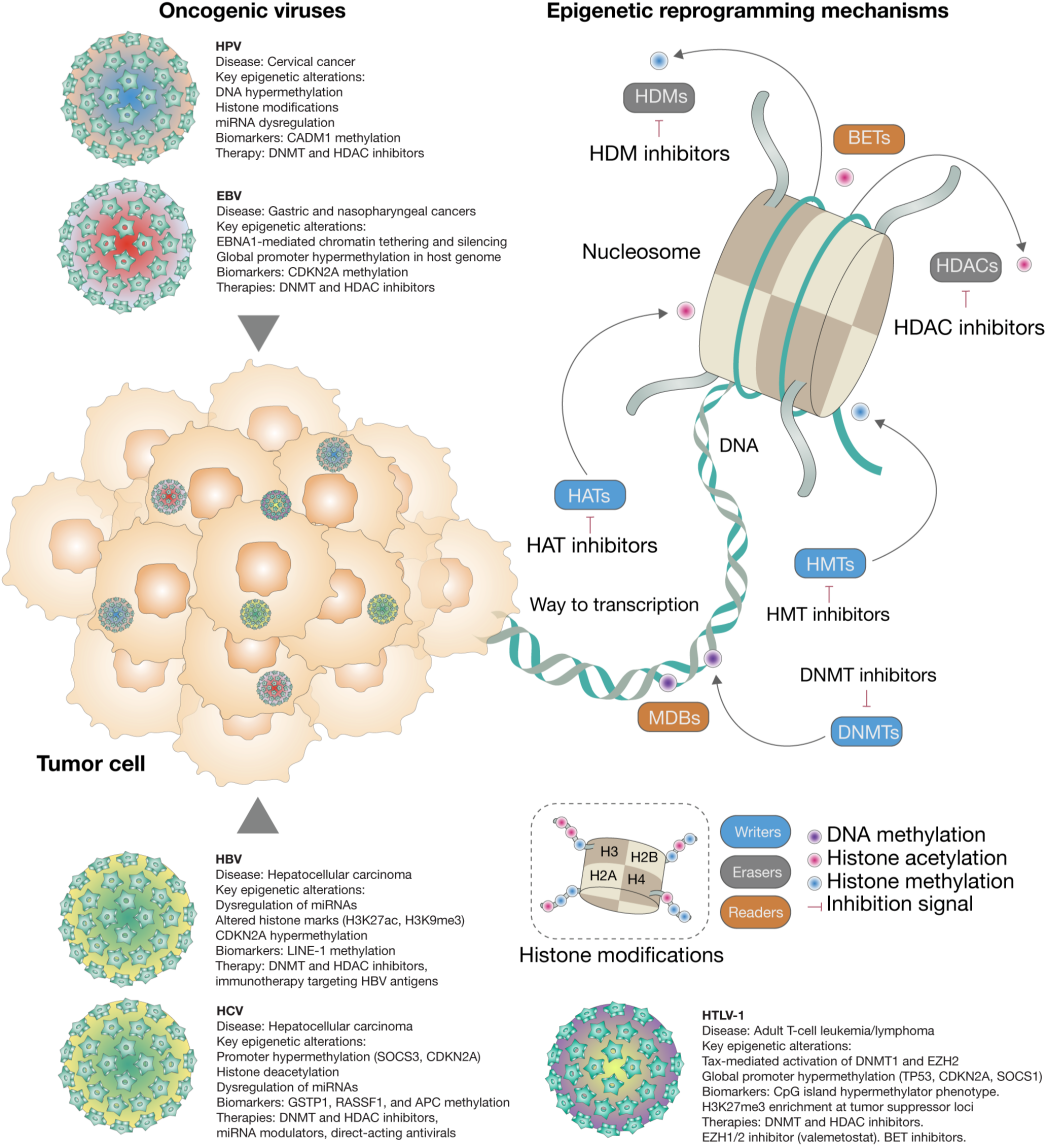
Epigenetic reprogramming by oncogenic viruses in human cancer. This schematic summarizes how oncogenic viruses such as HPV, EBV, HBV, HCV, and HTLV-1 reshape the host epigenome to promote tumorigenesis. Key viral proteins modulate chromatin accessibility and gene expression by altering DNA methylation, histone modifications, chromatin remodeling, and non-coding RNA expression. Enzymes such as DNA methyltransferases (DNMTs), histone methyltransferases (HMTs), histone acetyltransferases (HATs), histone deacetylases (HDACs), and demethylases (e.g., TETs, KDMs) dynamically write or erase epigenetic marks. These virus-induced epigenetic changes silence tumor suppressor genes, activate oncogenic pathways, and contribute to immune evasion and cancer progression—even after viral clearance.

Understanding the epigenetic consequences of viral oncogenesis provides not only mechanistic insights but also therapeutic opportunities. Epigenetic modifications are, by nature, reversible—offering a rationale for targeting virus-induced epimutations through pharmacological agents such as DNA methyltransferase inhibitors (DNMTis) and histone deacetylase inhibitors (HDACis)​ (Sultan et al., 2025). Furthermore, oncolytic virotherapy—viruses engineered to selectively kill cancer cells—has emerged as a promising approach, with evidence suggesting that these viruses can reprogram the tumor epigenome to enhance immunogenicity and sensitize tumors to treatment (Sultan et al., 2025; Faghihkhorasani et al., 2023). In this review, we explore the complex interplay between oncogenic viruses and host epigenetic reprogramming. We summarize current knowledge on how viral infections disrupt epigenetic regulation and how these changes contribute to tumorigenesis. Special emphasis is placed on innovative combined interventions—therapies that simultaneously target epigenetic dysregulation and immune evasion—highlighting their emerging role in overcoming treatment resistance and achieving durable cancer control (**Figure 1**).

## Interaction between human papillomavirus and epigenetic mechanisms in cervical cancer

Persistent infection with high-risk HPVs (HR-HPVs), particularly genotypes 16 and 18, is the major etiological factor in cervical cancer (CC). The oncogenic potential of HPV is driven largely by the sustained expression of viral oncoproteins E6 and E7, which reprogram host gene expression through both genetic and epigenetic mechanisms (Dueñas-González et al., 2005; Liu et al., 2023). E6 promotes the degradation of p53 via the E6AP ubiquitin ligase, while E7 inactivates the retinoblastoma protein (pRb), disrupting the G1/S cell cycle checkpoint (Narisawa-Saito and Kiyono, 2007). Beyond these classical targets, both oncoproteins are potent epigenetic modulators. E6 has been shown to upregulate DNA methyltransferases (DNMT1, DNMT3A, and DNMT3B) through both p53-dependent and independent pathways, leading to promoter hypermethylation of tumor suppressor genes (Cheng et al., 2019). Simultaneously, E7 interacts with HDACs and the Mi-2/NuRD repressor complex, enforcing a repressive chromatin state on genes involved in cell cycle control, apoptosis, and differentiation (Zhang et al., 2025; Soto et al., 2017; Poreba et al., 2011; Di Domenico et al., 2018).

### DNA methylation and silencing of tumor suppressors

One of the most studied epigenetic changes in HPV-transformed cells is hypermethylation of cell adhesion molecule 1 (*CADM1*), death-associated protein kinase 1 (*DAPK1*), and retinoic acid receptor beta (*RARB*) gene promoters (Laengsri et al., 2018). These changes, often mediated by DNMT overexpression, result in gene silencing that facilitates epithelial–mesenchymal transition (EMT) and immune evasion. Notably, cyclin-dependent kinase inhibitor 2A (*CDKN2A*) and L1 cell adhesion molecule (*L1CAM*) hypermethylation correlates with tumor progression and invasiveness (Weiss et al., 2022; Ogiwara and Kohno, 2012).

### Histone modifications and chromatin remodeling

HPV oncoproteins also disrupt histone modification landscapes. E7 recruits HDAC1/2 and EZH2, the methyltransferase component of PRC2, promoting repressive H3K27me3 marks on tumor suppressors​ (Soto et al., 2017; Liu et al., 2023; Durzynska et al., 2017). E6 suppresses p300/CBP HATs, lowering acetylation at H3 and H4, leading to condensed chromatin and silenced genes (Ibrahim et al., 2022).

### Non-coding RNAs in network rewiring

HPV infection induces extensive non-coding RNA (ncRNA) deregulation. It downregulates tumor-suppressive miRNAs like miR-34a, miR-143, and miR-145, affecting the PI3K/AKT and Wnt/β-catenin pathways (Ranga et al., 2023). These miRNAs are often silenced via epigenetic repression driven by E6/E7 (Lajer et al., 2012; Castro-Oropeza and Piña-Sánchez, 2022). Simultaneously, HPV upregulates oncogenic long non-coding RNA (lncRNAs) such as HOTAIR and MALAT1, which modulate chromatin modifiers and sponge tumor-suppressive miRNAs, reinforcing oncogenic signaling (Castro-Oropeza and Piña-Sánchez, 2022).

### Clinical implications

The reversibility of epigenetic modifications makes them attractive targets for therapy. Biomarkers like *CADM1* methylation are being tested in non-invasive screening and epigenetic drugs—such as DNMTis (e.g., 5-azacytidine; NTC01349959 and NCT01105377) and HDACis (e.g., romidepsin; NCT04639843)—are in preclinical and early-phase clinical trials (Dueñas-González et al., 2005; Poreba et al., 2011; Zhang et al., 2022; Li et al., 2014). Notably, targeting *EZH2* or restoring miR-34a can enhance the efficacy of immune checkpoint inhibitors, highlighting the promise of combined epigenetic–immunotherapeutic approaches (Tao et al., 2022).

### Epigenetic modulation by Epstein-Barr virus in associated cancers

EBV, a ubiquitous gammaherpesvirus, is implicated in the development of various lymphoid and epithelial malignancies including Burkitt lymphoma (BL), Hodgkin lymphoma (HL), nasopharyngeal carcinoma (NPC), and EBV-associated gastric carcinoma (EBVaGC). EBV’s oncogenic capacity is largely attributed to its profound ability to reprogram host epigenetic landscapes to promote viral latency, immune evasion, and cellular transformation (Shareena and Kumar, 2023; Leong and Lung, 2021; Scott, 2017). EBV persists in the host in either latent or lytic forms. During latency, viral gene expression is restricted and epigenetically regulated to avoid immune detection. The latency program includes the expression of genes such as *EBNA1*, *LMP1*, *LMP2A/B*, *EBERs*, and *BARTs*, depending on latency type (I–III). These genes not only maintain the viral episome but also manipulate host epigenetic mechanisms to modulate transcription of both viral and host genomes (Shareena and Kumar, 2023; Scott, 2017; Yau et al., 2014).

### DNA methylation and chromatin remodeling

EBV-infected cells exhibit marked promoter hypermethylation of tumor suppressor genes such as CDH1, PTEN, p16, DAPK, and RASSF1A. This is particularly evident in EBVaGC and NPC, which show a CpG island methylator phenotype (CIMP) characterized by dense promoter methylation across multiple gene loci (Niller et al., 2016; Cao et al., 2021; Yau et al., 2014). Latent proteins LMP1 and LMP2A drive these effects through the upregulation of *DNMT1*, *DNMT3A*, and *DNMT3B*, leading to transcriptional silencing of tumor suppressors (Yau et al., 2014; Scott, 2017; Okano, 2000).

### Histone modifications

Beyond DNA methylation, EBV also manipulates histone modifications. The viral oncoproteins (e.g., EBNA2, EBNA3C, LMP1) recruit histone-modifying complexes such as the polycomb repressive complex 2 (PRC2), resulting in the deposition of H3K27me3 and repression of critical genes involved in apoptosis and cell cycle control (Gequelin et al., 2011; Cao et al., 2021; Scott, 2017). In epithelial cancers, EBV was shown to upregulate KDM5B—a histone demethylase—through EBNA1 and BZLF1, contributing to the silencing of PLK2 and activation of the PI3K/AKT/mTOR pathway, as demonstrated in EBV-associated NPC and EBVaGC models (Zhou et al., 2025).

### Non-coding RNAs and miRNAs

EBV also encodes microRNAs, particularly from the BART and BHRF1 clusters, which function as epigenetic regulators. These miRNAs modulate viral and host gene expression by targeting transcripts such as *BIM*, *PUMA*, *DICER1*, and *LMP1* itself, thereby influencing cell survival and immune evasion (Yau et al., 2014). EBV miRNAs can also downregulate host tumor suppressor miRNAs like the Let-7 family, contributing further to epigenetic dysregulation (Yau et al., 2014).

### Host epigenetic reprogramming

EBV-mediated epigenetic reprogramming extends to permanent changes in host chromatin. EBV infection of epithelial cells (e.g., nasopharyngeal and gastric) results in long-lasting epigenetic alterations, even after loss of the viral genome. Studies using telomerase-immortalized oral keratinocytes and gastric epithelial models showed that EBV induces CIMP, delays differentiation, enhances invasiveness, and alters gene expression in ways that mimic cancer phenotypes (Yau et al., 2014; Scott, 2017).

### Disease-specific epigenetic signatures

The epigenetic profiles of EBV-associated cancers vary by tissue type. In BL, EBV-positive tumors show widespread DNA hypermethylation and lower mutational burden than their EBV-negative counterparts, suggesting that EBV epigenetic modulation may replace the need for genetic mutations (Scott, 2017). In HL, EBV induces a hypomethylation phenotype in germinal center B cells through differential expression of DNMT isoforms, contributing to the phenotype of Reed–Sternberg cells (Cao et al., 2021). In EBVaGC, EBV induces hypermethylation of critical tumor suppressor genes including *p16*, *APC*, and *PTEN*—hallmarks of this gastric cancer subtype (Yau et al., 2014).

### Therapeutic implications

Because EBV-induced epigenetic modifications are reversible, they represent attractive therapeutic targets. DNA methyltransferase inhibitors (e.g., decitabine, azacitidine) and histone deacetylase inhibitors (e.g., vorinostat, romidepsin) have shown efficacy in preclinical models of EBV-associated malignancies (Mabe et al., 2024; Ocaña-Paredes et al., 2024). Furthermore, inhibition of KDM5B with AS-8351 suppressed tumor growth in NPC xenografts, reinforcing the potential of targeting EBV-activated histone demethylases (Zhou et al., 2025).

## Influence of hepatitis B virus on epigenetic mechanisms in hepatocarcinogenesis

HBV infection remains a leading cause of hepatocellular carcinoma (HCC) worldwide, particularly in Asia and sub-Saharan Africa. Chronic HBV infection contributes to more than 50% of global HCC cases and over 80% in endemic regions, due in part to the virus’s ability to integrate into the host genome and disrupt regulatory networks through both genetic and epigenetic mechanisms (Zhang et al., 2024b; Tian and Ou, 2015; Tian et al., 2013).

A central player in this oncogenic process is the HBV-encoded X protein (HBx), which serves as a potent modulator of the host cell environment. HBx promotes hepatocarcinogenesis by inducing epigenetic aberrations such as DNA methylation, histone modification, and non-coding RNA dysregulation (Tian et al., 2013; Zhang et al., 2017). These changes affect key tumor suppressor and oncogene pathways, facilitating immune escape, chronic inflammation, and uncontrolled proliferation of hepatocytes.

### DNA methylation

One of the most extensively characterized epigenetic alterations in HBV-related HCC is the hypermethylation of CpG islands in tumor suppressor gene promoters, including *p16*, *RASSF1A*, *E-cadherin*, and *GSTP1*. HBx enhances the expression of DNA methyltransferases (*DNMT1*, *DNMT3A*, *DNMT3B*), promoting transcriptional silencing of these genes (Tian and Ou, 2015; Dandri, 2020). This process not only favors malignant transformation but also contributes to the maintenance of viral persistence by silencing immune regulatory genes (Farazi and DePinho, 2006).

### Histone modifications

HBx also disrupts histone post-translational modifications by recruiting or modulating histone-modifying enzymes, such as HDACs and histone methyltransferases. These interactions result in histone deacetylation or methylation at specific loci, leading to chromatin condensation and gene repression. For example, histone H3K9 and H3K27 trimethylation, linked to gene silencing, are enriched in HBx-expressing cells (Yang et al., 2022; Zhang et al., 2024b).

### Non-coding RNAs

HBV, particularly through HBx, alters the expression of miRNAs and lncRNAs, which serve as key regulators of gene expression. Several miRNAs downregulated by HBx (e.g., miR-122, miR-199a-3p) target oncogenes and signaling pathways such as Wnt/β-catenin, PI3K/AKT, and TGF-β, while others promote angiogenesis and epithelial–mesenchymal transition (EMT) (Levrero and Zucman-Rossi, 2016; Tian et al., 2013). Concurrently, dysregulated lncRNAs like HULC and HEIH act as competing endogenous RNAs, sponging tumor-suppressive miRNAs and driving HCC progression (Yang et al., 2022).

### HBV covalently closed circular DNA epigenetics

The viral covalently closed circular DNA (cccDNA) acts as a stable minichromosome within hepatocytes. HBx modulates the epigenetic status of cccDNA by recruiting histone acetyltransferases and methyltransferases, thereby regulating viral gene expression and latency. These modifications allow HBV to persist despite antiviral treatment and contribute to treatment resistance (Yang et al., 2022; Dandri, 2020).

### Immune and inflammatory modulation

Chronic HBV infection alters the epigenetic landscape of immune cells, impairing antiviral responses. HBx induces immunosuppressive environments through epigenetic reprogramming of cytokine genes and immune checkpoint molecules, enabling viral persistence and fostering a pro-tumorigenic inflammatory microenvironment (Levrero and Zucman-Rossi, 2016; Zhang et al., 2024b).

### Therapeutic implications

Targeting HBV-driven epigenetic alterations offers promising avenues for therapy. Agents such as DNMT inhibitors (e.g., azacytidine, decitabine) and HDAC inhibitors (e.g., entinostat, panobinostat) are being investigated for their capacity to reverse gene silencing and sensitize HCC cells to chemotherapy and immunotherapy (Lin et al., 2025; Tian et al., 2013). Moreover, modulation of non-coding RNAs and inhibition of epigenetic regulators affecting cccDNA may provide new strategies for viral clearance and HCC prevention (Dandri, 2020; Yang et al., 2022). Lastly, HBV promotes hepatocarcinogenesis through multilayered epigenetic modifications. HBx-mediated dysregulation of DNA methylation, histone modification, and non-coding RNA expression profoundly alters both viral and host gene networks. These insights underscore the significance of epigenetic therapies in managing HBV-associated liver cancer and emphasize the need for further mechanistic and translational research.

## Epigenetic mechanisms in chronic hepatitis C virus infection and its role in hepatic cancer

HCV is a hepatotropic, positive-sense RNA virus that infects more than 70 million people worldwide and remains one of the leading causes of HCC. Unlike DNA viruses, HCV does not integrate into the host genome; however, it contributes to hepatocarcinogenesis via persistent inflammation, immune evasion, and profound epigenetic remodeling of infected hepatocytes (Fiehn et al., 2024; Feng, 2013; Pan et al., 2024).

### Persistent epigenetic imprinting and HCC risk

HCV-associated HCC can arise even after viral clearance through direct-acting antivirals (DAAs), suggesting that infection leaves a durable epigenetic “scar” on hepatocytes. These alterations include long-lasting changes in histone marks (e.g., H3K27ac and H3K9me3), chromatin accessibility, and DNA methylation that dysregulate key cancer-related pathways such as Wnt/β-catenin and TGF-β signaling (García-Crespo et al., 2023; Hamdane et al., 2019). Genome-wide studies have shown that these HCV-induced epigenetic marks persist in patients with sustained virological response (SVR), potentially contributing to post-SVR HCC risk (Hamdane et al., 2019; Lohmann and Bartenschlager, 2019).

### DNA methylation alterations

Chronic HCV infection leads to both global hypomethylation and regional hypermethylation in promoter CpG islands. Tumor suppressor genes including RASSF1A, SOCS1, and CDKN2A are frequently silenced through DNA hypermethylation, while global hypomethylation contributes to genomic instability and aberrant gene expression (Zhao et al., 2021; Dash et al., 2020). These methylation patterns can be detected in circulating cell-free DNA, highlighting their potential as non-invasive biomarkers (Braghini et al., 2022; Pan et al., 2024).

### Histone modifications

HCV core and non-structural proteins (particularly NS5A) interact with chromatin modifiers such as HDACs and methyltransferases to alter histone marks. These changes can suppress immune-related genes and promote oncogene expression, establishing a pro-carcinogenic chromatin environment even in the absence of active viral replication (Zhao et al., 2021; Dash et al., 2020; Hamdane et al., 2019).

### Non-coding RNAs and epigenetic crosstalk

miRNAs and lncRNAs are significantly dysregulated in HCV-infected livers. For example, downregulation of tumor-suppressive miR-122 and upregulation of oncogenic lncRNAs such as HOTAIR and HULC are common in HCV-related HCC (Pan et al., 2024; Feng, 2013). These non-coding RNAs influence epigenetic machinery by targeting DNMTs, HDACs, and polycomb repressive complexes, reinforcing transcriptional silencing of tumor suppressors and enhancing EMT and stemness features (Feng, 2013; Braghini et al., 2022).

### Cancer stem cells and EMT

HCV-driven epigenetic changes promote a cancer stem cell–like phenotype by activating stemness pathways (Wnt, Notch, Hedgehog) and repressing differentiation-related genes. Epigenetic reprogramming facilitates EMT, enhancing invasiveness and metastatic potential (Pan et al., 2024; Zhao et al., 2021). Polycomb group proteins (e.g., EZH2) and histone demethylases are implicated in this transformation and are considered actionable targets (García-Crespo et al., 2023; Feng, 2013).

### Impact of DAA therapy

While DAA therapy achieves high SVR rates and reduces liver inflammation, recent studies reveal that epigenetic alterations established during chronic infection may not be reversed upon viral clearance. This may explain the persistent risk of HCC after SVR, especially in patients with advanced fibrosis or cirrhosis (Dash et al., 2020; Hamdane et al., 2019; Fiehn et al., 2024). Thus, understanding the durability and functional consequences of HCV-induced epigenetic changes is essential for post-therapy surveillance and risk stratification (Hamdane et al., 2019; Lohmann and Bartenschlager, 2019).

### Therapeutic and diagnostic implications

Epigenetic biomarkers are being investigated for early detection of HCV-related HCC, including methylated DNA loci and non-coding RNAs detectable in serum (Pan et al., 2024). Lastly, epigenetic drugs such as DNMT inhibitors (e.g., azacytidine) and HDAC inhibitors (e.g., vorinostat) are being explored for their capacity to reverse epigenetic silencing and restore immune responsiveness (Braghini et al., 2022; Zhao et al., 2021).

## Epigenetic reprogramming by human T-Cell leukemia virus type 1 in adult T-Cell leukemia

HTLV-1 is a deltaretrovirus responsible for adult T-cell leukemia/lymphoma (ATL), an aggressive malignancy of CD4^+^ T lymphocytes. Approximately 5% of infected individuals progress to ATL after a prolonged latency period, during which epigenetic dysregulation accumulates and contributes to transformation (Fujikawa et al., 2016; Xiao et al., 2025). Unlike other oncogenic viruses, HTLV-1 does not require site-specific integration or classical oncogenes for transformation. Instead, its main oncogenic driver, the Tax oncoprotein, reprograms the host epigenome to sustain proliferation, suppress apoptosis, and promote immune evasion (Yamagishi et al., 2019; Rosewick et al., 2017; Mohanty et al., 2024).

### Tax-mediated epigenetic remodeling

Tax plays a central role in ATL pathogenesis by modulating chromatin structure. It recruits histone-modifying enzymes and transcriptional coactivators to key gene loci. For example, Tax activates NF-κB signaling through KDR-dependent stabilization, which promotes chromatin remodeling and survival of infected T cells (Mohanty et al., 2024). Tax also upregulates histone methyltransferases like EZH2 and DNMT3B, leading to aberrant accumulation of H3K27me3 and hypermethylation of CpG islands at tumor suppressor loci including *CDKN2A*, *TP53*, and *SOCS1 (* Yamagishi et al., 2021 *;* Rosewick et al., 2017 *;* Matsuo et al., 2022 *)*. These modifications silence genes critical for cell cycle regulation and apoptosis. Furthermore, integrative genomic studies have shown that Tax disrupts global chromatin accessibility patterns, establishing repressive chromatin at differentiation genes and open chromatin at oncogenic enhancers (Lieberman, 2016). A novel enhancer within the HTLV-1 provirus itself has been identified as a regulatory hotspot driving persistent antisense transcription, further supporting the epigenetic basis of viral latency and transformation (Matsuo et al., 2022).

### Super-enhancer remodeling and transcription factor hijacking

A key discovery in HTLV-1 epigenetic oncogenesis is the remodeling of super-enhancers at transcription factor hubs. The HTLV-1-encoded HBZ protein binds a super-enhancer within the *BATF3* locus, amplifying a transcriptional program driven by BATF3 and IRF4—two master regulators of ATL proliferation. Disruption of this circuitry with BET inhibitors collapses the transcriptional network and suppresses ATL in preclinical models, highlighting its therapeutic relevance (Nakagawa et al., 2018).

### Non-coding RNA deregulation

HTLV-1 also reprograms the expression of non-coding RNAs. miR-31 and other tumor-suppressive microRNAs are downregulated through promoter hypermethylation, while oncogenic lncRNAs like *HOTAIR* and *ANRIL* are upregulated, contributing to immune evasion and proliferative advantage (Yamagishi et al., 2019; Xiao et al., 2025). These epigenetic changes in non-coding RNA expression further reinforce the leukemic phenotype.

### Therapeutic advances targeting epigenetic abnormalities

A major breakthrough in HTLV-1-related cancer therapy is the development of valemetostat, a dual EZH1/2 inhibitor. Valemetostat has demonstrated durable clinical responses in ATL by eliminating H3K27me3-enriched chromatin and reactivating silenced tumor suppressor genes (Yamagishi et al., 2024). Single-cell epigenomic analyses revealed that resistance emerges through compensatory DNA methylation mediated by elevated DNMT3A or TET2 mutations, underscoring the complexity of chromatin homeostasis in therapy resistance (Yamagishi et al., 2024). BET inhibitors, which disrupt enhancer function and transcription factor recruitment, have also shown efficacy in ATL xenografts and *ex vivo* models (Nakagawa et al., 2018). Their combination with HDACis is being explored to enhance therapeutic response and overcome resistance mechanisms (Yamagishi et al., 2024, Yamagishi et al., 2019). HTLV-1 drives ATL through multilayered epigenetic mechanisms including aberrant DNA methylation, histone modification, enhancer hijacking, and non-coding RNA deregulation. These changes are orchestrated primarily by the viral proteins Tax and HBZ and converge on the silencing of tumor suppressors and the amplification of oncogenic transcriptional programs. The approval and clinical success of epigenetic modulators such as valemetostat mark a turning point in ATL management and open the door to rational combination therapies targeting chromatin dynamics and transcriptional addiction in HTLV-1-induced malignancies (Mohanty et al., 2024; Fujikawa et al., 2016).

Lastly, the virus-specific epigenetic mechanisms of HPV, EBV, HBV, HCV, and HTLV-1 is summarized in **Table 1**.

**Table 1:** Virus-specific epigenetic mechanisms of HPV, EBV, HBV, HCV, and HTLV-1.

Virus	DNA Methylation	Histone Modifications	Non-coding RNAs	Chromatin Remodeling
HPV	Promoter hypermethylation of *CADM1*, *DAPK1*, *RARB* via DNMT1/3A/3B	E6 suppresses p300/CBP HATs; E7 recruits HDAC1/2 and EZH2 → H3K27me3	miR-34a, miR-143, miR-145 downregulated; HOTAIR, MALAT1 upregulated	Mi-2/NuRD complex and HDAC-mediated repression
EBV	CpG island methylator phenotype (CIMP); silencing of *CDH1*, *PTEN*, *RASSF1A* via DNMT1/3A/3B	EBNA2, LMP1 recruit PRC2 → H3K27me3; KDM5B upregulation	BART/BHRF1 viral miRNAs target tumor suppressors; Let-7 family suppressed	Persistent chromatin reprogramming in epithelial models
HBV	Hypermethylation of *p16*, *RASSF1A*, *GSTP1* via DNMT1/3A/3B	HBx modulates HDACs, HMTs → H3K9/27 trimethylation	Downregulates miR-122, miR-199a-3p; upregulates HULC, HEIH	HBx alters host and cccDNA chromatin status
HCV	Promoter hypermethylation (*SOCS1*, *CDKN2A*); global hypomethylation	NS5A and core proteins modulate HDACs and methyltransferases	miR-122 downregulated; HOTAIR, HULC upregulated; reinforces EMT/stemness	Long-lasting epigenetic marks even after SVR
HTLV-1	Hypermethylation of *TP53*, *CDKN2A*, *SOCS1* via DNMT1/3B; CpG island hypermethylator phenotype	Tax and HBZ promote EZH2 recruitment → H3K27me3; suppression of p300/CBP HATs; BET protein redistribution	Downregulation of miR-31; upregulation of ANRIL, HOTAIR; HBZ-regulated BATF3/IRF4 transcription network	Novel viral enhancer activates antisense transcription; super-enhancer remodeling by HBZ; persistent reprogramming of host chromatin

DNMT, DNA methyltransferase; HAT, Histone acetyltransferase; HDAC, Histone deacetylase; HMT, Histone methyltransferase; KDM, Histone demethylase; PRC2, Polycomb repressive complex 2; cccDNA, Covalently closed circular DNA; miRNA, microRNA; lncRNA, long non-coding RNA; EMT, epithelial–mesenchymal transition; SVR, sustained virological response; NF-κB, Nuclear factor kappa-light-chain-enhancer of activated B cells; ATL, Adult T-cell leukemia/lymphoma; HTLV-1, Human T-cell leukemia virus type 1. The arrow indicates a causal or functional consequence.

## Therapeutic approaches in oncogenic virus-induced cancers

Oncogenic viruses present unique therapeutic opportunities due to their distinct molecular signatures and immunogenic features. Treatments are evolving to target not only viral components and infected cells but also the epigenetic and immune alterations they induce. In this section, we explore virus-specific strategies—including immunotherapies, therapeutic vaccines, oncolytic virotherapy, drug repurposing, and epigenetic modulators—that are being developed or clinically tested to combat virus-driven malignancies (Ahmed and Jha, 2023; Vandeven and Nghiem, 2014; Yang et al., 2023; Krump and You, 2018).

### Virus-specific targeting strategies

Immunotherapies have emerged as powerful tools to treat virus-associated malignancies. Unlike conventional therapies, immune-based approaches can specifically distinguish infected from non-infected cells. Strategies include the adoptive transfer of virus-specific T cells (VSTs), checkpoint blockade, and dendritic cell (DC)-based vaccines. Clinical trials using EBV- and HPV-targeted T cells have shown durable responses in nasopharyngeal carcinoma, EBV-positive lymphomas, and cervical cancer (Tashiro and Brenner, 2017; Chakravorty et al., 2022). Moreover, adoptive cell therapies (ACTs) targeting virally encoded tumor antigens like EBNA1 (EBV), E6/E7 (HPV), HBx (HBV), and Tax (HTLV-1) are under exploration (Zhang et al., 2024a). In HTLV-1, Tax remains a key therapeutic target due to its role in maintaining leukemic cell survival through NF-κB activation and immune escape (Mohanty et al., 2024).

### Therapeutic vaccines and oncolytic viruses

**Figure 2:**
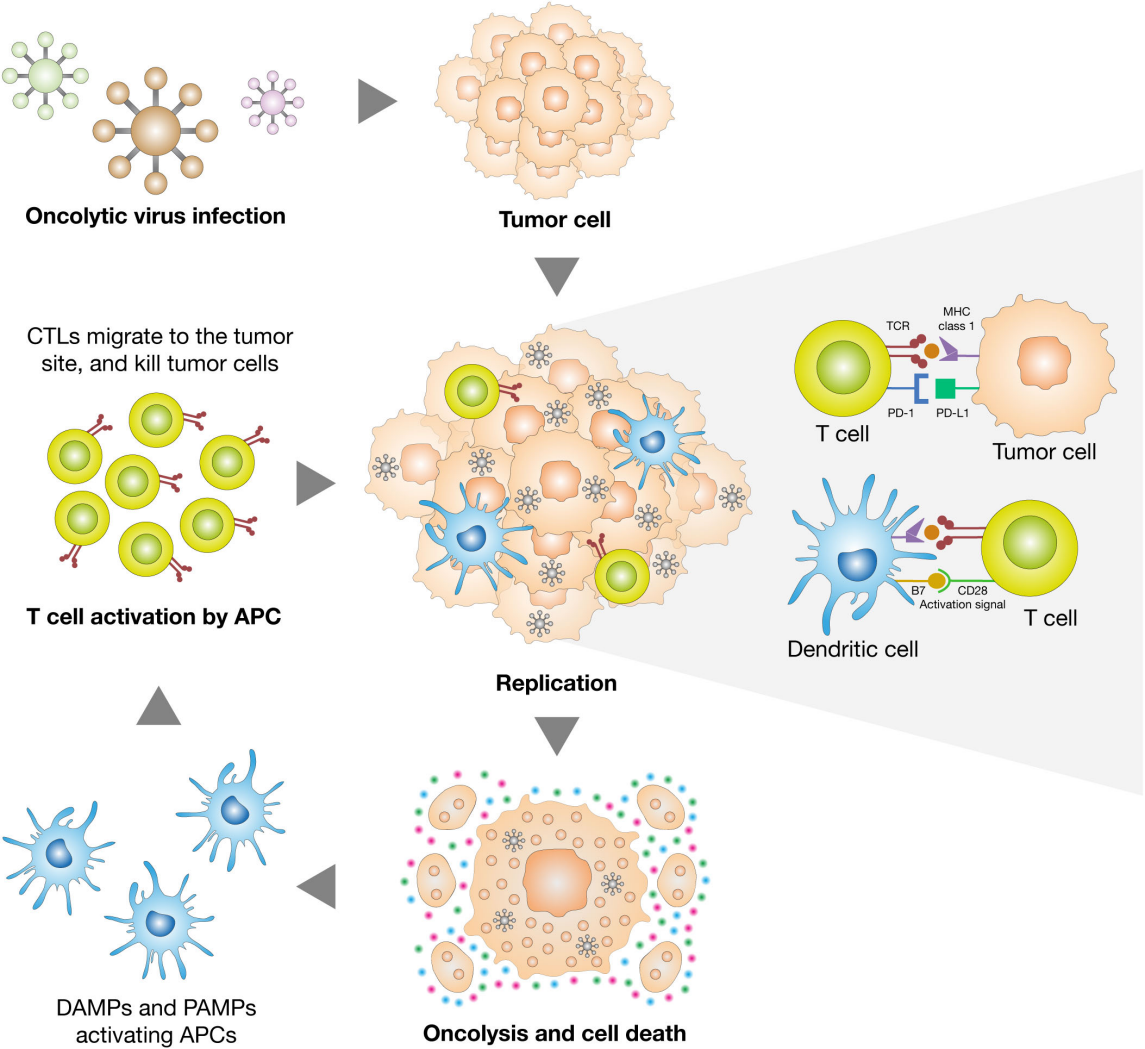
Dual mechanism of tumor elimination by oncolytic viruses. Oncolytic viruses exert anti-tumor effects through both direct and immune-mediated mechanisms. Upon selective infection of cancer cells, they replicate and induce immunogenic cell death, releasing danger-associated molecular patterns (DAMPs) and pathogen-associated molecular patterns (PAMPs). These signals activate dendritic cells (DCs), which present tumor antigens and prime cytotoxic T lymphocytes (CTLs). The activated CTLs then infiltrate the tumor microenvironment and mediate additional tumor cell killing, amplifying the therapeutic response.

Despite the availability of prophylactic vaccines for HPV and HBV, therapeutic vaccines aimed at eliciting robust cytotoxic T cell responses are under active investigation. However, their effectiveness is often dampened by the immunosuppressive tumor microenvironment. To overcome this, combination regimens involving therapeutic vaccines and metronomic chemotherapy—low-dose, frequent chemotherapeutic schedules—have been shown to enhance immunogenicity in HPV- and EBV-driven tumors (Zhang et al., 2024a; Faghihkhorasani et al., 2023; Kyriakidis et al., 2021; Ortiz-Prado et al., 2021). Additionally, oncolytic virotherapy represents a dual-function platform capable of lysing tumor cells while stimulating antiviral immunity (**Figure 2**). Herpesviruses and adenoviruses engineered to express immune stimulators are being explored in EBV- and HPV-positive tumors (Faghihkhorasani et al., 2023). In the case of HTLV-1, early-phase studies of peptide-based Tax vaccines and DC-Tax immunotherapy show potential to induce antitumor responses and reduce viral load in ATL patients, although clinical efficacy remains to be fully validated (Rosewick et al., 2017; Xiao et al., 2025).

### Drug repurposing and combination approaches

The unique biology of virus-induced cancers has driven efforts to repurpose non-cancer drugs (e.g., antivirals, anti-inflammatories) with known safety profiles. Metformin, statins, and NSAIDs have demonstrated anti-proliferative effects in HPV- and HBV-associated cancers through both metabolic and epigenetic reprogramming (Fernandes, 2024; Chowdhary et al., 2023; López-Cortés et al., 2021). In ATL, studies suggest that repurposed drugs that target NF-κB signaling or autophagy pathways—such as KDR inhibitors—may impair Tax stability and HTLV-1 cell survival (Mohanty et al., 2024). These drugs are being evaluated in combination with epigenetic therapies or immune checkpoint inhibitors to overcome resistance and improve outcomes (Fernandes, 2024; Chowdhary et al., 2023; Mohanty et al., 2024; Nakagawa et al., 2018).

### Antiviral Agents and Epigenetic Modulators

Current antiviral therapies effectively suppress viral replication (e.g., nucleos(t)ide analogs for HBV, direct-acting antivirals for HCV), but do not eliminate latent infections or reverse virus-induced epigenetic changes. In this context, epigenetic drugs such as DNMTis (e.g., azacitidine) and HDACis (e.g., vorinostat) are being evaluated for their ability to reactivate silenced immune genes and sensitize virus-driven tumors to immunotherapy (Ahmed and Jha, 2023; Wang et al., 2018; Chowdhary et al., 2023). Targeting viral miRNAs and host epigenetic regulators also holds promise for disrupting immune evasion strategies, particularly in EBV-driven cancers (Zhang et al., 2024a). In HTLV-1-associated ATL, the dual EZH1/2 inhibitor valemetostat has shown promising clinical activity, reversing H3K27me3-mediated silencing and restoring expression of pro-apoptotic and cell cycle regulators. Valemetostat was recently approved in Japan for relapsed/refractory ATL (Yamagishi et al., 2024). Additionally, BET inhibitors targeting BRD4-mediated transcriptional addiction in HTLV-1-transformed cells have shown preclinical efficacy and are being considered for combination strategies with HDACis or immunomodulators (Nakagawa et al., 2018; Matsuo et al., 2022).

### Personalized and precision approaches

Advances in omics and systems pharmacology are enabling the identification of virus-specific molecular signatures and actionable targets. In HPV-associated cancers, integrated transcriptomic and metabolomic analyses have identified natural compounds and phytochemicals capable of reversing E6/E7-induced immune evasion (Aarthy et al., 2022). Similarly, in HTLV-1, epigenomic profiling has uncovered enhancer landscapes and non-coding RNA networks (e.g., miR-31, HOTAIR, ANRIL) that may serve as predictive biomarkers or therapeutic targets (Xiao et al., 2025; Yamagishi et al., 2019). Single-cell ATAC-seq and methylome studies in ATL are aiding in stratifying patients by their epigenetic vulnerability to EZH2 or BET inhibition (Yamagishi et al., 2021, Yamagishi et al., 2024).

## Conclusions, challenges, and future perspectives

Oncogenic viruses are responsible for an estimated 15–20% of all human cancers globally, highlighting their profound impact on public health and cancer etiology (Ameya and Birri, 2023; Zapatka et al., 2020). These aforementioned viruses have evolved sophisticated mechanisms to manipulate host gene expression and cellular behavior through epigenetic reprogramming. Unlike somatic mutations, epigenetic changes are dynamic and reversible, offering both insight into the mechanisms of viral carcinogenesis and therapeutic opportunities (Poreba et al., 2011; Flanagan, 2007; Tashiro and Brenner, 2017).

Recent research has shown that viral oncoproteins can directly modulate the host epigenetic landscape by altering DNA methylation patterns, histone modifications, chromatin accessibility, and non-coding RNA expression. These alterations drive cancer hallmarks such as sustained proliferation, immune evasion, and resistance to apoptosis—even after the virus is cleared or enters latency (Tashiro and Brenner, 2017; Damian, 2025). For instance, persistent “epigenetic scars” have been documented in HPV-driven cervical cancer and HCV-associated hepatocellular carcinoma, explaining continued cancer risk after apparent viral clearance (Sultan et al., 2025; Aarthy et al., 2022). In the case of HTLV-1, the Tax and HBZ proteins induce widespread repressive chromatin changes and enhancer remodeling that drive ATL even decades after infection, underscoring the long latency of epigenetic reprogramming (Yamagishi et al., 2019; Nakagawa et al., 2018).

Emerging studies now highlight that virus-induced epigenomic alterations extend beyond linear chromatin modifications to include disruptions in 3D genome architecture. Viral genomes can reshape topologically associating domains (TADs), enhancer-promoter interactions, and nuclear compartmental organization, fundamentally altering spatial gene regulation in infected cells. For instance, Kim et al. demonstrated that EBV episomes persist in Burkitt lymphoma cells by attaching to host chromatin at specific genomic regions via the viral protein EBNA1, which tethers the episome to AT-rich regions enriched in H3K9me3, EBF1, and RBP-jκ binding sites. These EBV–host interaction sites are associated with transcriptionally repressed genes, including neuronal regulators and components of the protein kinase A signaling pathway. Notably, depletion of EBNA1 relieved this silencing and reduced H3K9me3 levels, suggesting that EBV chromatin tethering modulates host transcription in a latency type–specific manner (Kim et al., 2020). In parallel, Okabe et al. found revealed that in EBV-positive gastric cancer, non-integrated EBV episomes establish long-range physical contacts with host chromatin, remodeling repressive H3K9me3-marked domains into active enhancer-like states (marked by H3K4me1 and H3K27ac) at key proto-oncogenic loci such as *TGFBR2* and *MZT1*. This phenomenon, known as enhancer infestation, represents a novel oncogenic paradigm in which non-integrative viral episomes directly rewire 3D chromatin topology to activate tumor-promoting gene networks and facilitate transformation (Okabe et al., 2020). In parallel, HTLV-1 integrates into transcriptionally active loci and utilizes a newly discovered intragenic enhancer to maintain antisense transcription of *HBZ*, while Tax promotes redistribution of BET proteins and activation of oncogenic super-enhancers such as *BATF3*, reprogramming the transcriptional circuitry of infected T cells (Nakagawa et al., 2018; Matsuo et al., 2022).

Despite major advances in identifying epigenetic alterations and viral gene targets, critical challenges remain. One major limitation is the difficulty of distinguishing early epigenetic drivers of transformation from late-stage consequences in established tumors. Furthermore, the heterogeneity of virus-associated tumors—arising from distinct viral strains, latency programs, and tissue-specific responses—complicates therapeutic targeting (Flanagan, 2007; Faghihkhorasani et al., 2023). Latent viruses, such as EBV and HTLV-1, maintain oncogenic potential without producing viral particles, evading both immune surveillance and antiviral drugs (Damian, 2025; Szewczyk-Roszczenko et al., 2025). As a result, virus-induced tumors often exhibit resistance to conventional therapies and require more sophisticated, multimodal approaches.

Therapeutic strategies are rapidly evolving to address these complexities. Immunotherapy—particularly adoptive T cell transfer, VSTs, and checkpoint inhibitors—has shown encouraging results in treating EBV- and HPV-driven cancers (Tashiro and Brenner, 2017; Faghihkhorasani et al., 2023). Oncolytic virotherapy, which selectively replicates in and lyses tumor cells while inducing systemic antitumor immunity, is also emerging as a promising approach, especially in cancers harboring cancer stem cell–like properties (Flanagan, 2007; Sultan et al., 2025). Complementing these are epigenetic drugs, such as DNMTis, HDACis, and more recently, EZH1/2 inhibitors and BET inhibitors, which aim to reverse transcriptional silencing and resensitize virus-driven tumors to immune and cytotoxic therapies. Notably, valemetostat, a dual EZH1/2 inhibitor, has been approved in Japan for relapsed/refractory ATL and represents a milestone in translating epigenetic understanding into clinical benefit (Nakagawa et al., 2018; Yamagishi et al., 2024).

Looking ahead, several research directions are critical for improving outcomes in patients with virus-induced malignancies. First, there is a need for large-scale, longitudinal studies to map virus-specific epigenetic alterations during all stages of infection and transformation. These studies will help differentiate causal from passenger changes and identify biomarkers for early detection and risk stratification (Poreba et al., 2011; Zapatka et al., 2020). Second, integrated multi-omics and systems pharmacology approaches can elucidate complex virus–host interactions and facilitate the design of rational, multi-targeted therapies—including plant-derived bioactive compounds and immuno-epigenetic drugs (Aarthy et al., 2022; Szewczyk-Roszczenko et al., 2025). Third, public health initiatives must be strengthened to expand access to preventive vaccines and early screening programs, particularly in low- and middle-income countries where viral cancers are most prevalent (Damian, 2025; Ameya and Birri, 2023). Equally important is the development of therapeutics that address the latent phase of viral infections and their long-term epigenetic impact, particularly in populations cured of infection but still at risk of malignancy. In conclusion, the convergence of virology, epigenetics, and immunotherapy offers an unprecedented opportunity to transform the clinical management of virus-induced cancers. As our understanding of viral epigenomics deepens, the field is poised to deliver not only novel insights into carcinogenesis but also transformative therapeutic solutions tailored to the unique biology of virally driven tumors.
